# Pseudothrombotic Microangiopathy as a Rare Presentation of Cobalamin Deficiency

**DOI:** 10.7759/cureus.17184

**Published:** 2021-08-14

**Authors:** Carla Pereira Fontes, Samuel Fonseca

**Affiliations:** 1 Internal Medicine, Centro Hospitalar de Entre o Douro e Vouga, Santa Maria da Feira, PRT

**Keywords:** cobalamin deficiency, pseudo-thrombotic microangiopathy, pernicious-anemia, schistocytes, pancytopenia

## Abstract

The hematological manifestations of cobalamin (vitamin B12) deficiency may range from asymptomatic to life-threatening forms. Pseudothrombotic microangiopathy is a rare but severe presentation, characterized by the presence of hemolysis and schistocytosis, that is completely reversible after vitamin supplementation.

We present a challenging diagnostic approach of a 55-year-old man who presented with high hemolytic markers, pancytopenia, and schistocytes on the peripheral smear due to acquired cobalamin deficiency. Subsequent testing revealed positive anti-intrinsic factor and anti-parietal cell antibodies consistent with pernicious anemia. Cobalamin replacement led to the resolution of microangiopathic hemolysis and clinical improvement, thereby confirming the diagnosis.

This case highlights the importance of early recognition of this syndrome, which is often misdiagnosed as true microangiopathic hemolytic anemia, confounding appropriate management.

## Introduction

Cobalamin, or vitamin B12, is a complex water-soluble vitamin synthesized by microorganisms present mainly in animal products. A regular non-vegetarian diet usually contains an adequate portion of cobalamin (3-30 µg/day) that sustains the typical daily requirement (2-5 µg) [1]. Body stores of cobalamin are primarily hepatic and are in the range of 2-5mg, hence its deficit develops slowly, manifesting over several years (average of five to ten years) [1-3]. Classically, the diagnosis is based on a low cobalamin level, determined by the WHO as less than 150 pmol/L (203 pg/mL) on two different occasions [4].

Epidemiological studies show a 20% prevalence of cobalamin deficiency in the general population of industrialized countries. In elderly and/or institutionalized subjects, the prevalence may be higher, ranging from 12% to 30-40% [5]. However, due to the lack of a consensual definition, these numbers vary according to the threshold of normality used and, thus, real prevalence is difficult to assess.

The major causes of cobalamin deficiency are malabsorption (secondary to pernicious anemia, reduced gastric acidity, gastrointestinal pathology, or surgery) and inadequate dietary intake associated with vegetarianism [5,6]. Other etiologies include hereditary metabolic diseases, fish tapeworm infection, and exposition to drugs that interfere with cobalamin absorption or stability [7,8].

Cobalamin depletion adversely affects neuronal function and hematological lineages. Therefore, its main clinical manifestations are neuropsychiatric and hematological. The severity is highly variable, from mild to severe conditions. Many patients exhibit signs of peripheral nervous system or spinal cord involvement, including polyneuritis (particularly sensitive and distal), ataxia, dementia, delirium, or psychosis [1, 3, 7]. Several life-threatening hematological disorders have been reported in cobalamin deficiency cases, such as symptomatic pancytopenia (5%), severe anemia with hemoglobin levels less than 6 g/dL (2.5%), pseudothrombotic microangiopathy (2.5%), and hemolytic anemia (1.5%) [9]. The severity of all these changes is generally proportional to the degree of anemia.

We report a rare case of pseudothrombotic microangiopathy in the setting of cobalamin deficiency, resolved after vitamin supplementation.

## Case presentation

A 55-year-old male, without significant previous medical or surgical history, presented to the emergency department with complaints of progressive fatigue and anorexia over the past three months. He denied fever, abdominal pain, or bleeding. There were no medications, drugs, or dietary restrictions in the patient's history. There was also no family history of anemia or any other hematological disorder.

On physical exam, the patient was afebrile, hemodynamically stable, pale, and anicteric; no hepatosplenomegaly, rash, or palpable purpura was observed. He did not exhibit any stigmata of bleeding and had a normal rectal examination. There were no neurological deficits and the examination of the remaining systems was normal.

Laboratory investigation revealed anemia (hemoglobin of 5.5 g/dL), leukopenia (3.200/μL), and thrombocytopenia (60.000/μL). Prominent anisopoikilocytosis with teardrop cells, schistocytes, and hypersegmented neutrophils were present on the peripheral blood smear. His metabolic panel showed normal transaminases, a mildly elevated indirect bilirubin (3.02 mg/dL), a markedly elevated lactate dehydrogenase (2211 U/L) level, and a diminished haptoglobin (<8 mg/dL), pointing towards significant hemolysis. Erythrocyte indices indicated macrocytosis (mean corpuscular volume of 113 fL), associated with a low corrected reticulocyte count (1.25%). Coombs (antiglobulin) tests were negative. His hematinics showed a severely low serum cobalamin level (<83 pg/mL) with normal folate levels and iron stores. The remaining analyses, including thyroid parameters, renal function, and electrolytes, were normal. The test for HIV was negative.

In this case, despite initial doubt of a more serious condition, since the patient remained hemodynamically stable with no evidence of active hemorrhage, vascular disturbance, or organ impairment, the hypothesis of a secondary form of microangiopathy due to severe cobalamin deficiency prevailed. The concomitant presence of leukopenia, macrocytosis, and an inadequate reticulocyte response to the degree of anemia supported this suspicion.

The patient was transfused immediately with two units of packed red blood cells with an adequate response (hemoglobin 7.1 g/dL).

Once the diagnosis of cobalamin depletion was established, the patient was discharged with once-weekly administration of 1 mg intramuscular cobalamin.

Further investigation revealed serum anti-intrinsic factor and anti-parietal cell antibodies supporting a diagnosis of pernicious anemia. 

At the 2-month follow-up, the patient was asymptomatic and his condition improved noticeably with sustained normalization of blood counts, disappearance of schistocytes on the peripheral smear, and resolution of the microangiopathic hemolysis, thereby confirming the diagnosis.

## Discussion

Most cases of cobalamin deficiency present only mild hematological findings; rarely, severe conditions such as pseudothrombotic microangiopathy have been described, as illustrated in this report. Alternative or concomitant etiologies for anemia and hemolysis were sought and ruled out; however, as myeloproliferative disorders were less likely, a bone marrow biopsy was not performed. The effective response to supplementation assured confidence to diagnosis and excluded additional investigations.

The combination of thrombocytopenia, anemia and schistocytosis should raise suspicion of thrombotic microangiopathy and a thorough search for secondary causes should be performed. Differential diagnosis includes potentially life-threatening associated conditions such as thrombotic thrombocytopenic purpura (TTP), hemolytic uremic syndrome, or disseminated intravascular coagulation.

Pseudothrombotic microangiopathy closely resembles the clinical features of TTP, therefore, distinguishing between the two is of paramount importance, as the management, treatment, and prognosis are markedly different. Unlike severe thrombocytopenia associated with TTP, the average platelet count of patients with pseudothrombotic microangiopathy is usually higher (approximately 70.000 platelets/μL). Acute kidney injury and severe neurological symptoms are common in TTP, but rare in pseudothrombotic microangiopathy [9,10].

The pathogenesis of this syndrome is poorly understood. Immune dysfunction has been questioned since the majority of cases are associated with pernicious anemia [11]. Some literature suggests that high levels of homocysteine and/or its derivatives may be a trigger to endothelial dysfunction, which results in the fragmentation of erythrocytes [12]. Cobalamin deficiency leads to defective DNA synthesis and, consequently, the production of macrocytes with reduced deformability and lower hemoglobin concentration (megaloblastic cells) that get entrapped in the microcirculation [13,14]. This intramedullary hemolysis results in higher lactate dehydrogenase levels, but relatively modest rises in unconjugated bilirubin compared to peripheral hemolysis of mature erythrocytes seen in TTP. Reticulocytopenia is secondary to the abnormal maturation process (ineffective erythropoiesis), differing from other hemolytic anemias in which an undamaged marrow responds with remarkable reticulocytosis [10,15].

Standard treatment consists of intramuscular cobalamin replacement once monthly for life, preceded by an initial intensive regimen (1mg weekly for eight weeks or 1mg daily for seven days followed by once weekly for four weeks); an equally effective alternative for maintenance therapy involves higher doses of oral cobalamin (1-2mg daily) in compliant patients [5,16].

## Conclusions

Pseudothrombotic microangiopathy can be challenging for accurate diagnosis and appropriate management due to its low incidence and similarities to true microangiopathic hemolytic anemia. Thus, clinicians should be aware of patients at risk of cobalamin deficiency and extend differential diagnoses based on clinical manifestations and biochemical markers.
